# Effects of In Utero Exposure to Di-n-Butyl Phthalate on Testicular Development in Rat

**DOI:** 10.3390/ijerph14101284

**Published:** 2017-10-24

**Authors:** Tan Ma, Xiaoqin Yin, Ruitong Han, Jie Ding, Huan Zhang, Xiaodong Han, Dongmei Li

**Affiliations:** 1 Immunology and Reproduction Biology Laboratory & State Key Laboratory of Analytical Chemistry for Life Science, Medical School, Nanjing University, Nanjing 210093, China; MTlLYJ@163.com (T.M.); yinxiaoqin1991@126.com (X.Y.); naruto18f7@163.com (R.H.); djie@nju.edu.cn (J.D.); 2 Jiangsu Key Laboratory of Molecular Medicine, Nanjing University, Nanjing 210093, China; 3 Department of Clinical and Experimental Medicine, Linköping University, SE-581 83 Linköping, Sweden

**Keywords:** prenatal DBP exposure, testicular cells, ras related dexamethasone induced 1 (Rasd1), MEK1/2, Bcl-2, Bax, cell proliferation, apoptosis

## Abstract

Humans are inevitably exposed to ubiquitous phthalate esters (PAEs). In utero exposure to di-n-butyl phthalate (DBP) induces abnormal development of the testis and reproductive tract in male offspring, which correspond closely with the human condition of testicular dysgenesis syndrome (TDS)-like syndrome. However, the underlying mechanisms have not been elucidated in detail. In this study, pregnant rats were orally exposed to either corn oil (controls) or DBP at three different doses by gavage during Gestational Days 12.5–21.5. Pathological examinations were performed for toxicity evaluation. Proliferation and apoptosis related proteins (ras related dexamethasone induced 1 (Rasd1), mitogen-activated protein kinase kinases1/2 (MEK1/2), Bcl-2, and Bax) were measured for mechanisms exploration. The results showed that different doses of DBP caused male developmental and reproductive toxicity in rats, including the decrease of anogenital distance (AGD), the histological damage of testis, and apoptosis of seminiferous tubule cells. Our data suggested that DBP played chronic and continuous toxic roles on male reproductive system by disrupting expression of Rasd1 and MEK1/2 as well as Bcl-2/Bax ratio. Further research is warranted.

## 1. Introduction

Over the past decades, male reproductive problems, such as poor spermatogenesis, testicular cancer, hypospadias, and cryptorchidism, are widespread in all parts of the World [1]. Evidence was presented that several adult male reproductive problems arise in utero and were signs of testicular dysgenesis syndrome (TDS) [2,3]. Although TDS might result from genetic mutations, recent evidence suggested that it most often was related to environmental exposures of the fetal testis; for example, many of the characteristics of the syndrome could be reproduced in the male offspring of female rats exposed to chemicals with estrogenic or anti-androgenic properties during pregnancy, such as di-n-butyl phthalate (DBP) [4].

Phthalate esters (PAEs) are industrial chemicals used primarily as plasticizers to impart flexibility to polyvinylchloride plastics. They are present in a wide variety of products, such as children’s toys, cosmetics, personal care products, food contact applications, and medical devices [5,6]. They are metabolized in the gut to the corresponding monoester and alcohol [7,8], with toxicity ascribed to the monoester metabolite [9,10].

DBP has been the most widely used PAEs for polyvinylchloride worldwide. Administration of the DBP to female rats at a dose of 500 mg/kg/day during pregnancy have been shown to interfere with normal development of reproductive organs in male offspring, which correspond closely with a human TDS-like condition [11,12], including underdevelopment or absent reproductive organs, malformation of the external genitalia, cryptorchidism, decreased anogenital distance (AGD), diminished sperm counts, Leydig cell adenomas [11], and also inducing an approximately 40% reduction in Sertoli cell (SC) numbers at the end of gestation [13,14]. Studies revealed that anti-androgenic effects was the mechanism of DBP leading to a variety of reproductive malformations in postnatal male rats after in utero exposure to DBP [15,16], while it was demonstrated that DBP has no affinity for androgen receptor in transcriptional activation assays [17]. Therefore, the mechanism by which DBP causes testicular developmental anomalies is still largely unknown, especially DBP long-term effects on reproductive function after male rats are in utero exposed to DBP [18].

The purpose of the present study was to explore the underlying mechanisms of DBP disturbing testicular development, based on confirming the developmental toxicity of DBP on reproductive system of male offspring from birth to adulthood following maternal exposure to DBP. In this study, pregnant rats were orally exposed to either corn oil (controls) or DBP at three different doses by gavage during Gestational Days (GD) 12.5–21.5. Pathological examinations were performed for toxicity evaluation. Proliferation and apoptosis related proteins (ras related dexamethasone induced 1 (Rasd1), mitogen-activated protein kinase kinases1/2 (MEK1/2), Bcl-2, and Bax) were measured for mechanisms exploration. The study, by offering a better understanding of DBP-induced developmental and reproductive toxicity, may provide useful information for understanding the mechanism of DBP disrupting male reproduction.

## 2. Materials and Methods

### 2.1. Ethics Statement

The animal experiments were performed according to the Guide for the Care and Use of Laboratory Animals (The Ministry of Science and Technology of China, 2006) and all experimental protocols were approved under the animal protocol number SYXK (Su) 2014-0052 issued by Jiangsu Provincial Science and Technology Department.

### 2.2. Chemicals

Di-n-butyl phthalate (DBP) and corn oil were purchased from Sigma-Aldrich Inc. (St. Louis, MO, USA). Other chemicals were of analytical grade.

### 2.3. Animals and Experimental Treatments

Nine-week-old male Sprague-Dawley (SD) rats (*n*_1_ = 4) and female rats (*n*_2_ = 12) were purchased from the Animal House of Nanjing Medical University, Nanjing, China. The advantages of using SD rats in the chemical toxicity testing include the fact that they share similarities with humans in terms of metabolic pathways and a number of physiological characteristics, in addition to the ease of handling, breeding and maintenance. Animals, housed in polycarbonate cages with chip hardwood animal bedding, were identified by tail tattoo, and were allowed ad libitum access to feed and filtered tap water. All animals were housed in an air-conditioned room at temperature 20–22 °C and humidity 50–70% with controlled lighting (12 h of light and 12 h of darkness) for 10 days prior to experiments and were fed with a pellet diet and water.

After 10 days of acclimatization, the female rats were housed overnight with the male rats. The day sperm was detected in the vaginal smear was considered GD 0.5. Pregnant rats were distributed into dose groups using body weight randomization and were treated from GD 12.5 to GD 21.5 with: 0 (control), 50, 250, or 500 mg/kg/day in 1 mL/kg corn oil administered daily by oral gavage. A previous study demonstrated that an oral dose of 500 mg/kg/day of DBP to the rat dam during GD 14–18 would be approximately half of the total effective dose which produces a 50% incidence (ED_50_) of epididymal agenesis [19]. Therefore, the highest concentration was 500 mg/kg/day.

After offspring were weighed and sexed at birth, males were kept together per dam. Weaning was carried out at 21 days postpartum, and pups were then removed from the mothers. Offspring were housed in polycarbonate cages with wood chips as bedding, which was replaced every 5 days; were fed a conventional diet (MF, Oriental Yeast, Osaka, Japan); and had free access to food and water. For each time point (at Postnatal Day (PND) 9, 21, 35, and 90), twelve males (three males from each cage representing each dose group were randomly selected) were removed, anesthetized, and euthanized by inhalation of CO_2_ followed by cervical dislocation. The AGD for male pups was measured by a digital micrometer on PND 9. The testes were carefully removed and in part of representative samples fixed in 4% paraformaldehyde and other part store at −80 °C.

### 2.4. Histopathological Analysis

Testes from the control and treated groups were removed and fragments selected from the distal segment of the gland were fixed by immersion in PBS for 24 h. Fixed tissue samples were dehydrated in a graded ethanol series and immersed in dimethylbenzene until transparency and embedded in paraffin. Histological sections (5 μm) were subjected to hematoxylin–eosin (H&E) staining for general studies. After H&E staining, the sections were mounted with neutral balsam for observation under a microscope DXM12000F (Nikon, Tokyo, Japan).

### 2.5. Immunohistochemistry

Testes were fixed in 4% paraformaldehyde solution for 24 h, embedded in paraffin and cut into 5-μm-thick sections. The sections were incubated with PBS containing 3% H_2_O_2_ for 10 min to inhibit endogenous peroxidase activity. After being blocked with 3% BSA in PBS for 1 h at 37 °C, the sections were subsequently incubated with rabbit anti-MEK, or anti-Rasd1 antibodies overnight at 4 °C using the dilutions listed in Table 1 and then with HRP-conjugated secondary antibodies (Boster) at 37 °C for 1 h. HRP activity was examined using the diaminobenzidine method according to the manufacturer’s instructions (Zhongshan Biotechnology, Beijing, China). After counterstaining with hematoxylin, the sections were mounted with neutral balsam for observation under a microscope DXM12000F (Nikon, Tokyo, Japan).

### 2.6. Western Blotting

One hundred milligrams of tissue were homogenized for extracting proteins in 1 mL ice-cold RIPA buffer (50 mM Tris-HCL pH 7.4, 150 mM NaCl, 1% NP, 0.1% SDS, 1 × phosphatase/protease inhibitors). The protein concentration was determined using the BCA protein assay kit (Beyotime, Nantong, China). Western blotting was performed as described previously [20]. About 20 μg of protein from each sample was separated on 10% SDS-PAGE and electrophoretically transferred to a polyvinylidene fluoride (PVDF) membrane. The membrane was then blocked in TBS buffer containing 5% bovine serum albumin (20 mM Tris–HCl (pH 7.6) and 150 mM NaCl) for 1.5 h at 37 °C. Next, these blots were incubated overnight at 4 °C with rabbit anti-GAPDH, rabbit anti-MEK1/2, rabbit anti-p-MEK1/2, rabbit anti-Bax, rabbit anti-Bcl-2, and rabbit anti-Rasd1 using the dilutions listed in Table 1. Blots incubated with rabbit IgG served as negative controls. The blots were then incubated with species-matched horseradish peroxidase-conjugated secondary antibodies (Absin, Shanghai, China). The chromogenic signal intensity was detected using an Odyssey Scanning System (LI-COR, Lincoln, NE, USA) and quantified using image J software (NIH, Bethesda, MD, USA).

### 2.7. Statistical Analyses

SPSS 18.0 (SPSS, Chicago, IL, USA) was used for statistical analysis. Experimental results were expressed as mean ± standard deviation. The Levene’s test was employed to check the normality and homogeneity of variances in the data. The Student’s *t* test was used for paired comparisons. For the comparison of three or more groups, one-way ANOVA was used, which was followed by Duncan’s post hoc test. Values of *p* < 0.05 were considered statistically significant.

## 3. Results

### 3.1. Associations between DBP Exposure and AGD in Offspring Males

**Figure 1:**
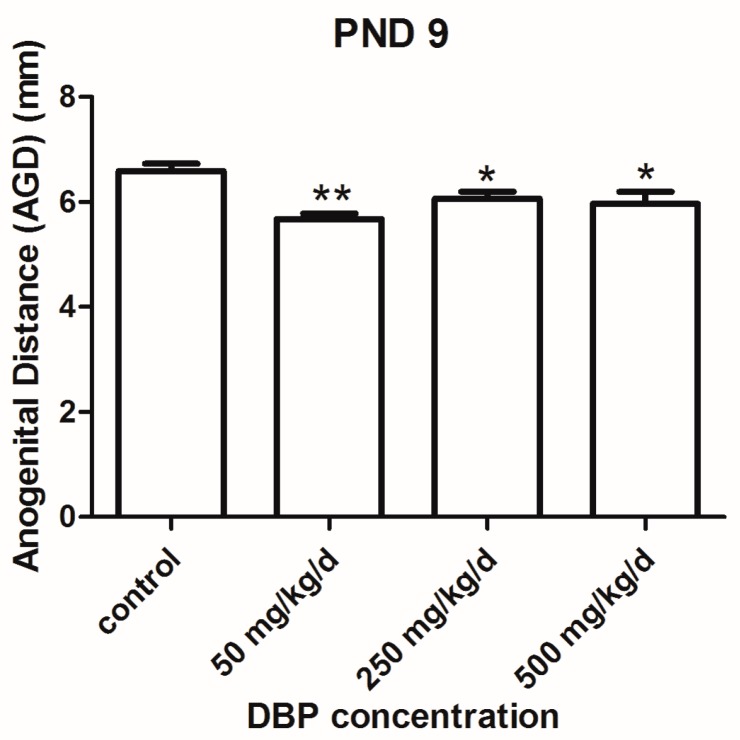
Effect of prenatal exposures to vehicle (oil) or to di-n-butyl phthalate (DBP) on anogenital distance (AGD) at Postnatal Day (PND) 9. Each bar is the mean ± SEM. ** *p* < 0.01; * *p* < 0.05, in comparison with control group. PND: Postnatal Day.

DBP was dosed by gavage at 50, 250, and 500 mg/kg/day (GD 12.5–21.5) in pregnant rats. We examined the pups of the DBP exposure at PND 9. The AGD in all DBP treated groups was significantly lower than that of the vehicle group (Figure 1).

### 3.2. Effect of DBP on Histologic Structure of Testis

**Figure 2:**
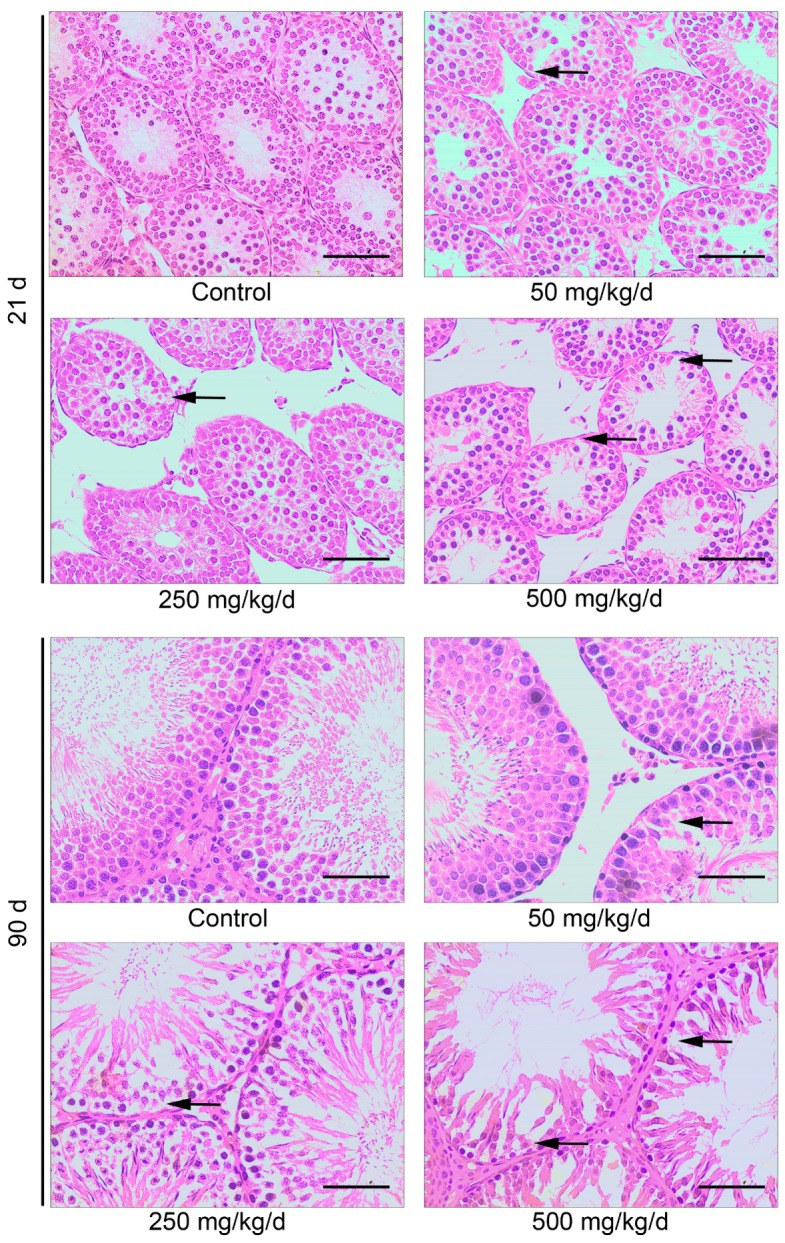
The impact of DBP on histologic structure of testis was determined by hematoxylin-eosin (H&E) staining. The arrows indicate the loss of cells in the seminiferous tubules. Scale bar = 100 μm.

We detected histological changes of the testes by H&E at different time. Figure 2 shows representative images of testicular cross section of all experimental groups, including seminiferous tubules at stages I–VIII [21]. The layers in the seminiferous tubules are organized from external to internal as basal lamina, spermatogonia, spermatocyte, and spermatid. These layers are readily distinguishable in testes from control rats. There was relatively slight damage to the testicular tissue in the group treated with 50 mg/kg/day. Obvious injury of the testicular tissue, characterized by severe atrophy and vacuoles of the seminiferous tubules, the spermatogenic epithelium becoming loosened in its organization and loss of spermatogenesis, was observed in the group treated with 250 mg/kg/day and 500 mg/kg/day (Figure 2). In offspring adult male rats, the seminiferous tubules of the testis were dilated in all treated groups, relative to controls. It was also observed the significant increase in the interstitial component in relation to tubular component in the gonads compared to control animals (Figure 2). As shown in Figure 2, tubules that contained germ cells frequently exhibited abnormal or reduced spermatogenesis, characterized by a decreased number of cells.

### 3.3. Effects of DBP on Bax and Bcl-2 Protein Expression

**Figure 3:**
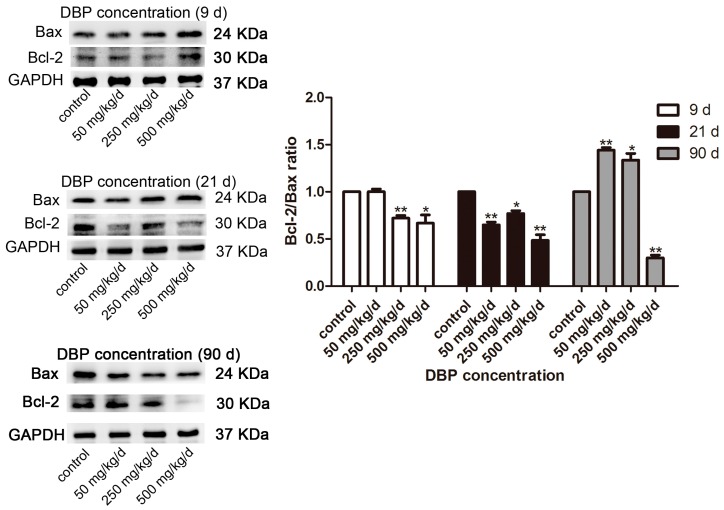
DBP induces the activation of pro-apoptosis protein in testicular tissue. The protein levels of Bax and Bcl-2 in testicular tissue treated with various concentration of DBP were measured by Western blot. The expression levels were quantified with ImageJ (right panel; *n* = 3). Glyceraldehyde-3-phosphate dehydrogenase (GAPDH) was run as an internal control. Results are expressed as means ± S.D. ** *p* < 0.01; * *p* < 0.05.

The number of testicular cells decreased with the increase of DBP concentration (Figure 2). To survey the effect of in utero exposure to DBP on testicular cells apoptosis, we analyzed the protein expression of some apoptosis-associated genes in rat testes following DBP treatment. The apoptotic index (Bcl-2/Bax ratio) was significantly decreased at PND 9 and 21, and, in 500 mg/kg/day DBP, groups at PND 90 (Figure 3). However, Bcl-2/Bax ratio increased significantly in 50 and 250 mg/kg/day DBP groups at PND 90 (Figure 3). These results suggested that in utero exposure to DBP could induced testicular cell apoptosis in male offspring, and cell proliferation can be restored with age increasing in the low and medium dose groups.

### 3.4. Effects of DBP on MEK1/2 Protein Expression

**Figure 4:**
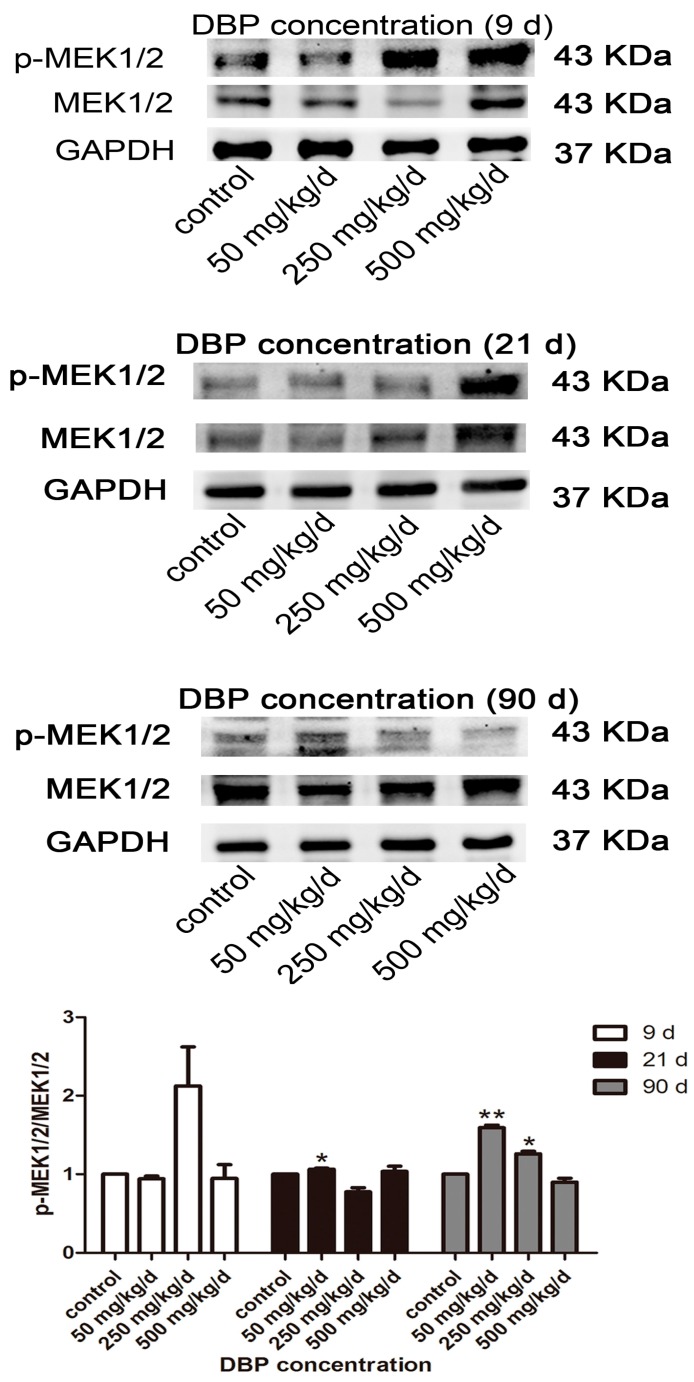
DBP induces the activation of mitogen-activated protein kinase (MAPK)-associated proliferation protein in testicular tissue. The protein levels of mitogen-activated protein kinase kinases1/2 (MEK1/2) and phospho-MEK1/2 (p-MEK1/2) in testicular tissue treated with various concentration of DBP were measured by Western blot. The expression levels were quantified with ImageJ (lower panel; *n* = 3). GAPDH was run as an internal control. Results are expressed as means ± S.D. ** *p* < 0.01; * *p* < 0.05. Scale bar = 100 μm.

To further explore the effect of DBP on cell growth, the key proliferation gene of MEK1/2 from MAPK/ERK pathway was chosen. As shown in Figure 4, phosphorylation of MEK1/2 increased significantly in 50 and 250 mg/kg/day DBP groups at PND 90, consistent with the results of Bcl-2/Bax ratio in 50 and 250 mg/kg/day DBP groups at PND 90 (Figure 3).

### 3.5. Effects of DBP on Rasd1 Protein Expression and Immunohistochemistry Analysis

**Figure 5:**
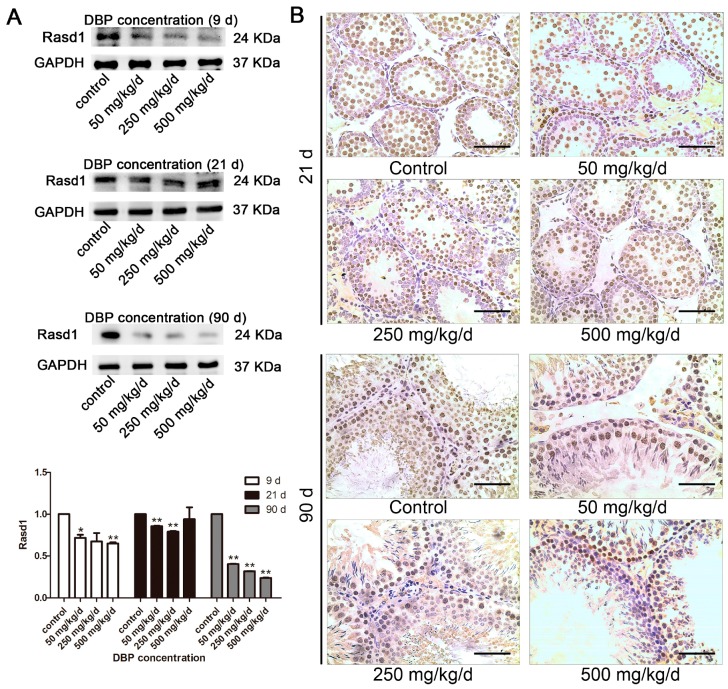
The effects of DBP on Rasd1 analysis: (**A**) The protein level of Rasd1 in testicular tissue treated with various concentrations of DBP were measured by Western blot. The expression of Rasd1 was quantified with ImageJ (lower panel; *n* = 3). GAPDH was used as a loading control; (**B**) Rasd1 in rat testicular tissue was revealed by immunohistochemistry. Results are expressed as means ± S.D. ** *p* < 0.01; * *p* < 0.05. Scale bar = 100 μm.

In our previous report, we performed mRNA and miRNA microarrays with total RNAs isolated from primary cultured Sertoli cells harvested on 24 h after 0.1 mM MBP treatment in vitro. The mRNA and miRNA microarray data were deposited to GEO (Gene Expression Omnibus) database [22]. Based on the microarray data, Rasd1 was chosen for further investigation because Rasd1 might play an important role in cell proliferation. At all time points, Rasd1 expression in the DBP groups was remarkably lower than control (Figure 5A). These results were also supported by immunohistochemical staining (Figure 5B).

## 4. Discussion

The ubiquity of phthalate esters in the environment makes the reported adverse effects on male reproductive development a matter of concern. Several previous studies have shown that in utero exposure to DBP leads to diminished testosterone production by the fetal rat testis [23,24]. However, phthalates, such as DBP, do not directly bind to androgen receptors, but exert direct effect(s) on rat male offspring [25,26]. Therefore, the purpose of the present study was to explore the underlying mechanisms whereby maternal exposure to DBP during gestation disturbed testicular development in her male offspring from birth to adulthood.

AGD, the distance between the anus and the genitals, is frequently 50–100% longer in males than females of many species, including humans. It is an alternative biomarker of fetal testicular function, which in animal models reflects androgen action during the masculinization programming window (MPW), the developmental period during which sufficient androgen exposure must occur to ensure subsequent normal differentiation and growth of male reproductive organs [27,28]. A shorter AGD in males has been associated with cryptorchidism; hypospadias; less masculine play behavior in childhood; and poor semen quality, infertility, small testes and low serum testosterone levels in adulthood [29,30]. For that reason, AGD, as well as testicular descent, and weight and structure of the external genitalia, is included in the United State Environmental Protection Agency (USEPA) guidelines for evaluation of chemicals for reproductive toxicity. In the present study, AGD was found to be inversely associated with DBP exposure. The reduction in AGD in male offspring rats after gestation exposed to DBP is presumed to be secondary result from DBP decreasing testicular testosterone production [31,32].

The histopathological injuries of testis were observed in this study. Testicular cell maturation was affected by DBP exposure, and the number of cells were reduced for collection points in a time and dose-dependent manner. In the damaged testis, the spermatogenic cells may enter disordered cell cycle progression, which is controlled by several key proteins, such as p53, Chk1, Cdc2, and CDK6 [33,34]. An ex vivo study showed that DBP (10^−6^ M, 24 h) could increase p53 expression in rat and mouse osteoblasts [35]. Other studies showed that DBP (100 mM, 24 h) could cause spermatogenic cell apoptosis by disrupting vimentin filaments in rat sertoli cells [36,37]. Moreover, Bcl-2 and its closest relatives (Bcl-xL, Bcl-w) promote cell survival, whereas Bax is identified as Bcl-2-interacting protein that opposed Bcl-2 and promotes apoptotic cell death [38]. Down-regulating the ratio of Bcl-2/Bax could induce apoptosis [39]. DBP (10^−8^–10^−6^ M, 24 h) decreased the ratio of Bcl-2/Bax in induced pluripotent stem cells, thereby favoring apoptosis [40]. In addition, Zhu et al. showed that activation of the MAPK/ERK signaling pathway, including MEK1/2 and extracellular signal regulated kinase 1/2 (ERK1/2) resulted in promoting MC3T3-E1 cell proliferation [41]. In the current study, we found that phosphorylation level of MEK1/2 and Bcl-2 expression was down-regulated, while Bax expression increased at PND 9 and 21. The ratio of Bcl-2/Bax was decreased, indicating that in utero exposure to DBP could induce apoptosis of testicular cell. However, phosphorylation level of MEK1/2 and Bcl-2 expression increased and the expression of Bax protein was reduced by exposure to 50 and 250 mg/kg/day DBP at PND 90, implying that the balance between cell proliferation and apoptosis was gradually restored with the increase of age.

The Ras dexamethasone-induced protein (Rasd) family members include Rasd1 and Rasd2, which share 62% identity [42]. Rasd1 has been known to play a role as a cell-signaling regulator of steroid hormone in several cells and tissues [43,44]. In addition, the expression of Rasd1 is also regulated by several steroid hormones [45,46]. Glucocorticoid and dexamethasone induced the expression of Rasd1 in AtT20 cells and corticotroph of pituitary [43,45], whereas glucocorticoid and prolactin regulated Rasd1 expression in pancreatic B-cells [44]. Interestingly, Rasd1 expression was induced by endogenous estrogen in mouse pituitary [43]. Previous reports suggested that the oocyte maturation was arrested via the knockdown Rasd1 gene [47]. Some other opinions concern the effect of changes in expression of Rasd1 on cell growth. Vaidyanathan et al. [45] showed that the Ras-related protein AGS1/RASD1 suppresses cell growth and tumor expansion, play an active role in preventing aberrant cell growth. Moreover, Takesono et al. have reported that Rasd1 transfection blocked GPCR-mediated regulation of G-protein-regulated inwardly rectifying potassium (GIRK) channels and activation of MAPK/ERK1/2 pathway [48]. It was shown that Rasd1 is highly expressed in reproductive organs including uterus, ovary, and testis, implying that Rasd1 is a cellular signaling regulator in the reproductive organ including the testis [49]. However, the role of Rasd1 regulating testicular cell proliferation was unknown. In the present study, the results showed that in utero exposure to DBP decreased the expression of Rasd1 in testis of male offspring, especially at PND 90. We speculated that down-regulated expression of Rasd1 may be one of the secondary results from DBP decreasing testicular testosterone production; thus, down-regulated Rasd1 may further promote cell proliferation by increasing phosphorylation of MEK1/2 in in utero exposure to 50 and 250 mg/kg/day DBP groups at PND 90.

## 5. Conclusions

In conclusion, our study demonstrated that in utero exposure to DBP inhibited cell proliferation and promoted the apoptosis of cells in the seminiferous tubules in the male offspring from birth to PND 21, although the balance between cell proliferation and apoptosis was gradually restored with the increase of age in the low and medium dose groups. In addition, it should be noted that the toxicological effects of DBP on development were chronic and continuous, and one of potential risks is that down-regulated Rasd1 may promote cell excessive proliferation by increasing phosphorylation of MEK1/2 in the low and medium dose groups. Therefore, more attention should be paid to this potential issue.

## Figures and Tables

**Table 1:** Antibodies used for Western blot (WB) and Immunohistochemistry (IHC) analysis.

Antibody	Species	Company	Catalog	Dilution
Anti-MEK	Rabbit Polyclonal Ab	Absin	abs120553	1/500 (WB) 1/100 (IHC)
Anti-p-MEK	Mouse mAb	CST	#9121s	1/1000 (WB)
Anti-Rasd1	Rabbit Polyclonal Ab	Proteintech	12634-1-AP	1/1000 (WB) 1/200 (IHC)
Anti-Bax	Rabbit Polyclonal Ab	Proteintech	50599-2-Ig	1/1000 (WB)
Anti-Bcl-2	Rabbit Polyclonal Ab	Proteintech	12789-1-AP	1/1000 (WB)
Anti-GAPDH	Mouse mAb	Absci	#AB44031	1/1000 (WB)
